# HMGB1 as a potential biomarker and therapeutic target for severe COVID-19

**DOI:** 10.1016/j.heliyon.2020.e05672

**Published:** 2020-12-07

**Authors:** Ruochan Chen, Yan Huang, Jun Quan, Jiao Liu, Haichao Wang, Timothy R. Billiar, Michael T. Lotze, Herbert J. Zeh, Rui Kang, Daolin Tang

**Affiliations:** a Department of Infectious Diseases, Xiangya Hospital, Central South University, Changsha, Hunan 410008, China; b Hunan Key Laboratory of Viral Hepatitis, Xiangya Hospital, Central South University, Changsha, Hunan 410008, China; c The Third Affiliated Hospital, Guangzhou Medical University, Guangdong 510600, China; d Laboratory of Emergency Medicine, North Shore University Hospital, Feinstein Institute for Medical Research, Manhasset, New York 11030, USA; e Department of Surgery, University of Pittsburgh, Pittsburgh, Pennsylvania 15219, USA; f Department of Surgery, UT Southwestern Medical Center, Dallas, Texas, USA

**Keywords:** HMGB1, COVID-19, Cell culture, Cell death, Inflammation, Infectious disease, Immunology, Microbiology, Virology

## Abstract

COVID-19 has attracted global attention due to its rapid spread around the world with substantial morbidity and associated mortality. Severe COVID-19 can be complicated by the acute respiratory distress syndrome, sepsis and septic shock leading to death. These complications are thought to result from an overactivation of the immune system, leading to a cytokine storm syndrome associated with multiple organ failure. Here, we report that high mobility group box 1 (HMGB1), a prototypical damage-associated molecular pattern (DAMP) and a central mediator of lethal inflammation, could be a potential target for innovative therapeutic strategies for COVID-19. Serum HMGB1 in severe COVID-19 patients is elevated (189.40 ± 140.88 ng/ml). Exogenous HMGB1 induces the expression of SARS-CoV-2 entry receptor ACE2 in alveolar epithelial cells in an AGER-dependent manner. Importantly, genetic (using AGER siRNA) or pharmacological (using glycyrrhizin, chloroquine, hydroxychloroquine, and FPS-ZM1) inhibition of the HMGB1-AGER pathway blocks ACE2 expression. Thus, HMGB1 inhibitors are likewise promising drug candidates for the treatment of patients suffering from COVID-19.

## 1 Introduction

In the past 20 years, humans have suffered from three coronaviruses outbreaks, including severe acute respiratory syndrome coronavirus (SARS-CoV) in 2003, Middle East respiratory syndrome coronavirus (MERS-CoV) in 2012, and the ongoing outbreak of the coronavirus disease 2019 (COVID-19) caused by a new virus SARS-CoV-2 [1]. SARS-CoV-2 is genetically closely related to SARS-CoV and appears to have a similar natural host (bat) and a human host receptor (angiotensin I-converting enzyme 2 [ACE2]), although there is some controversial information on intermediate hosts (e.g., snakes or pangolins) [2, 3, 4]. In humans, SARS-CoV-2 is mainly spread by droplets generated when an infected person coughs, sneezes, or exhales. The World Health Organization has officially declared COVID-19 as a pandemic, requiring an internationally coordinated response for prevention and treatment [5].

Severe COVID-19 can be complicated by the acute respiratory distress syndrome, sepsis and septic shock, leading to death [6]. These complications are thought to result from an overactivation of the immune system, leading to a cytokine storm syndrome associated with multiple organ failure [7]. Damage-associated molecular pattern molecules (DAMPs) are endogenous ‘alarmin’ molecules that are released from dead or damaged cells, activating the immune system by interacting with several pattern recognition receptors, including toll-like receptors (TLR2, TLR4, and TLR9) and the advanced glycosylation end-product specific receptor (AGER, also known as RAGE) [8, 9].

High mobility group box 1 (HMGB1) is a prototypical DAMP and a central mediator of lethal inflammation in infection and tissue damage [10, 11, 12]. Although HMGB1 is considered as a potential target for the treatment of COVID-19 [13, 14, 15, 16, 17, 18, 19], the changes and direct pathological effects of HMGB1 in COVID-19 are still poorly understood. In this study, we provide evidence that HMGB1 could be a biomarker and therapeutic target for severe COVID-19.

## 2 Materials and methods

### 2.1 Assay reagents

The antibodies to ACTB (#8227) and AGER (#ab172473) were purchased from Abcam. The antibody to TLR4 (#MAB14782) was purchased from R&D System. Glycyrrhizin (#CDS020796), chloroquine (#PHR1258), hydroxychloroquine (#H0915), FPS-ZM1 (#553030), and LPS (#L4130) were purchased from Sigma. Human reduced HMGB1 protein (#1690-HMB) was purchased from R&D System (purity: >95%; endotoxin level: <0.10 EU per 1 μg). HMGB1 (#NBP2-62766) and TNF (#DY210) ELISA kits were purchased from Novus Biologicals and R&D System, respectively. The coefficient of variability (%CV) between sample replicates of HMGB1 and TNF ELISA kits were 3.8 and 2.9, respectively.

### 2.2 Patient samples

All 40 patients with confirmed COVID-19 admitted to Xiangya Hospital from January 24 to April 10 were included in our study (Table 1). This study was approved by the ethics committee of Xiangya Hospital. Blood samples from patients with COVID-19 were collected at the time of admission (n = 40) before initiation of treatment and the day of the last blood draw before they discharged from hospital (n = 4). COVID-19 was confirmed by nucleic acid testing of SARS-CoV-2 in throat swab samples. The disease severity of all hospitalized COVID-19 patients was assessed on admission, according to the Seventh Version of the Novel Coronavirus Pneumonia Diagnosis and Treatment Guidance of China. The severe case was defined according to the following criterion: 1. Respiratory distress with a respiratory rate over 30 per minute; 2. Pulse oximeter oxygen saturation ≤93% in the resting state while breathing ambient air; 3. Arterial blood oxygen partial pressure (PaO2)/oxygen concentration (FiO2) ≤ 300 mmHg (1 mmHg = 0.133 kPa). Sepsis was established according to the Third International Consensus Definitions for Sepsis and Septic Shock (Sepsis-3) [20].

**Table 1:** Demographics, clinical features, and laboratory index of patients with COVID-19.

	Total (n = 40)	Non-severe (n = 29)	Severe (n = 11)	P-value
Age (years)	52.95 ± 19.28	45.37 ± 18.66	65.29 ± 12.33	<0.001
BMI	25.48 ± 2.75	25.93 ± 2.73	24.32 ± 2.59	0.472
Sex				
Male	19 (47.50%)	13 (44.83%)	6 (54.55%)	0.595
Underlying disease	12 (30.00%)	7 (24.14%)	5 (45.45%)	0.024
Fever	20 (50.00%)	12 (41.38%)	8 (72.73%)	0.035
cough	15 (37.50%)	10 (34.48%)	5 (45.45%)	0.080
Shortness of breath	10 (25.00%)	3 (10.34%)	7 (63.64%)	<0.001
Adverse outcome	2 (5.41%)	0 (0.00%)	2 (18.76%)	<0.001
Treatment				
Lopinavir/Ritonavir	8 (20.00%)	4 (13.79%)	4 (36.36%)	
α-interferon	2 (5.00%)	2 (6.90%)	0 (0.00%)	
α-interferon + Lopinavir/Ritonavir	9 (22.50%)	3 (10.34%)	6 (54.55%)	
Arbidol	3 (7.50%)	2 (6.90%)	1 (9.09%)	

### 2.3 Cell culture

Calu-3 (#HTB-55, a human lung epithelial cell line), HepG2 (#HB-8065, a human liver cell line), Caco2 (#HTB-37, a human intestine cell line), RT4 (#HTB-2, a human urinary bladder cell line), and THP1 (#TIB-202, a human monocytic cell line) cell lines were obtained from the American Type Culture Collection. The TLR4^−/−^ THP1 cell line (#thpd-kotlr4) was obtained from InvivoGen. These cells were grown in Eagle's Minimum Essential Medium, McCoy's 5a, or RPMI-1640 medium with 10% fetal bovine serum, 2 mM of L-glutamine, and 100 U/ml of penicillin and streptomycin. Since ACE2 can be expressed in various tissues, including lung, small intestine, testis, liver and kidney, Calu-3, HepG2, Caco2 and RT4 cell lines were used to analyze the effect of HMGB1 on ACE2 expression. THP1 cells were used to determine the effect of HMGB1 on TNF release.

### 2.4 RNAi

TARGETplus SMART pool siRNAs against the indicated genes (TLR4 [#L-008088-01-0005] and AGER [#L-003625-00-0005]) were purchased from Dharmacon. This pool was a mixture of four siRNAs provided as a single reagent. The Lipofectamine™ 3000 transfection reagent (#L3000001, Thermo Fisher Scientific) was used to deliver siRNAs into cells according to the manufacturer's instructions.

### 2.5 qPCR

Total RNA was extracted using a QIAGEN RNeasy Plus Kit according to the manufacturer's instructions. First-strand cDNA was synthesized from 1 μg of RNA using the iScript cDNA Synthesis kit (#1708890, Bio-Rad). Briefly, 20-μl reactions were prepared by combining 4 μl of iScript Select reaction mix, 2 μl of gene-specific enhancer solution, 1 μl of reverse transcriptase, 1 μl of gene-specific assay pool (20×, 2 μM), and 12 μl of RNA diluted in RNase-free water. Quantitative real-time PCR was carried out using synthesized cDNA, primers, and SsoFast EvaGreen Supermix (#172-5204, Bio-Rad). The data were normalized to *RNA18S* and the fold change was calculated via the 2^−ΔΔCt^ method [21, 22]. Relative concentrations of mRNA were expressed in arbitrary units based on the untreated group, which was assigned a value of 1.

### 2.6 Western blot analysis

Cells were lysed in Cell Lysis Buffer (#9803, Cell Signaling Technology) with protease inhibitor cocktail (Promega), phosphatase inhibitor cocktail (Sigma), and 1 mM Na_3_VO_4_. Cleared lysates were resolved by SDS-PAGE (#3450124, Bio-Rad) and then transferred onto PVDF membranes (#1704273, Bio-Rad) [23]. The membranes were blocked with Tris-buffered saline Tween 20 (TBST) containing 5% skim milk for 1 h at room temperature and then incubated with the indicated primary antibodies (1:1000–1:3000) overnight at 4 °C. After being washed with TBST, the membranes were incubated with an HRP-linked secondary antibody for 1 h at room temperature. The membranes were washed three times in TBST and then visualized and analyzed with a ChemiDoc Touch Imaging System (#1708370, Bio-Rad). The intensities of bands were analyzed with Image Lab software.

### 2.7 Statistical analysis

All data met the assumptions of the tests (e.g., normal distribution). Unpaired Student's *t* tests were used to compare the means of two groups. One-way analysis of variance (ANOVA) was used for comparison among the different groups. When the ANOVA was significant, *post hoc* testing of differences between groups was performed using the least significant difference test. A two-tailed *P* value <0.05 was considered statistically significant.

## 3 Results

### 3.1 Enhanced HMGB1 blood levels in COVID-19 patients

**Figure 1:**
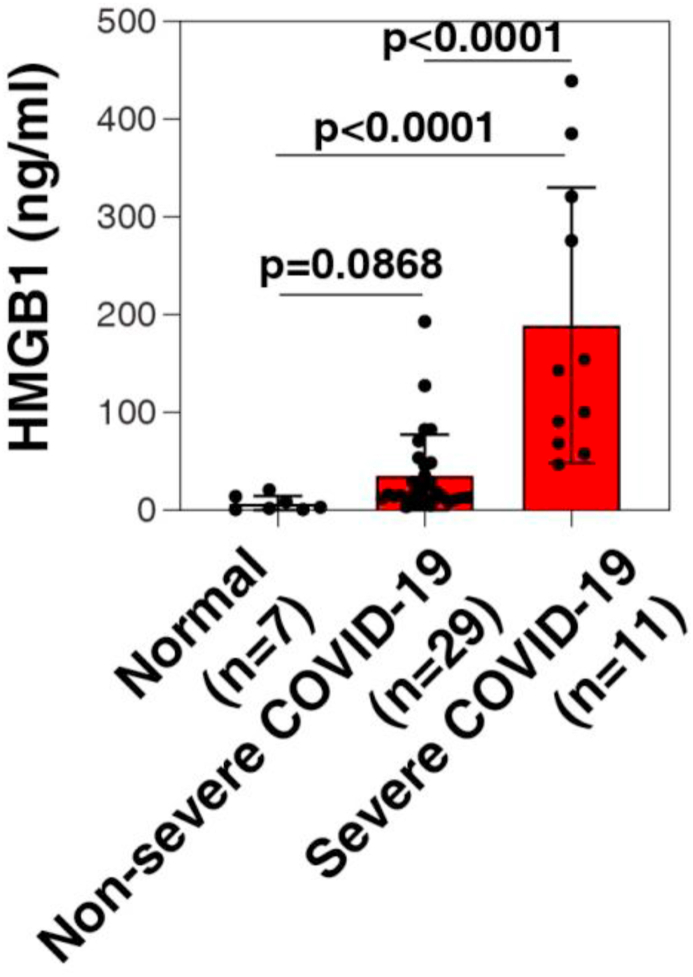
**Enhanced HMGB1 blood levels in COVID-19 patients.** ELISA analysis of the serum HMGB1 level in severe COVID-19 patients, non-severe COVID-19 patients, and healthy controls (*t* test).

We examined the serum HMGB1 levels of 11 severe and 29 non-severe COVID-19 patients following admission to Xiangya Hospital. The mean age of severe patients was 65.29 years (range, 25–74 years), and they were older than non-severe patients. Fever and shortness of breath are more common in severe patients or, which are typical symptoms of COVID-19 [6] and all had bilateral patchy shadows or ground glass opacity in the lungs on chest computed tomographic scans. More underlying diseases were found in severe patients, compared to non-severe patients. An significantly decreased lymphocyte count, increased D-dimer level (1.39, 0.18–6.39 mg/L; normal: 0.00–0.05 mg/L), as well as increased erythrocyte sedimentation rate (68.00, 46.00–86.00 mm/h; normal: 0.00–40.00 mm/h), lactate dehydrogenase (275.44 ± 97.12 U/L; normal: 0.00–250.00 U/L), and C-reactive protein (54.53, 31.60–70.67 mg/L; normal: 0.00–8.00 mg/L) were observed in severe patients (Table 2), indicating immunologic abnormalities. Importantly, the average serum HMGB1 level in the severe group was 189.40 ± 140.90 ng/ml, which was significantly higher than 35.51 ± 41.92 ng/ml in the non-severe group and 7.16 ± 2.88 ng/ml in normal control group (Figure 1). The information gathered from these case studies indicates that serum HMGB1 in COVID-19 patients positively correlated with disease severity. Serum HMGB1 was also decreased when patients improved, as detected in four severe patients (43.58 ± 37.02 ng/ml).

**Table 2:** Laboratory data of patients with COVID-19.

	Normal Range	Total (n = 40)	Non-severe (n = 29)	Severe (n = 11)	P-value
**Blood routine**					
Leucocytes, 10⁹/L	3.5–9.5	4.78 ± 1.72	4.73 ± 1.47	4.93 ± 2.41	0.913
Lymphocytes, 10⁹/L	1.1–3.2	1.17 (0.76–1.62)	1.27 (0.95–1.70)	0.64 (0.46–0.95)	<0.001

**Biochemical index**					
D-Dimer, mg/L	0–0.5	0.86 (0.14–1.47)	0.21 (0.14–0.42)	1.39 (0.18–6.39)	<0.001
Erythrocyte sedimentation rate, mm/h	0–40	40.50 (14.25–66.50)	33.00 (13.00–55.00)	68.00 (46.00–86.00)	0.007
High-sensitivity C-reactive protein, mg/L	0–8	11.10 (3.04–32.82)	5.95 (2.28–15.34)	54.53 (31.60–70.67)	<0.001
Lactate dehydrogenase, U/L	0–252	185.29 ± 78.52	158.40 ± 46.25	275.44 ± 97.12	<0.001

### 3.2 Recombinant human HMGB1 induces ACE2 expression

**Figure 2:**
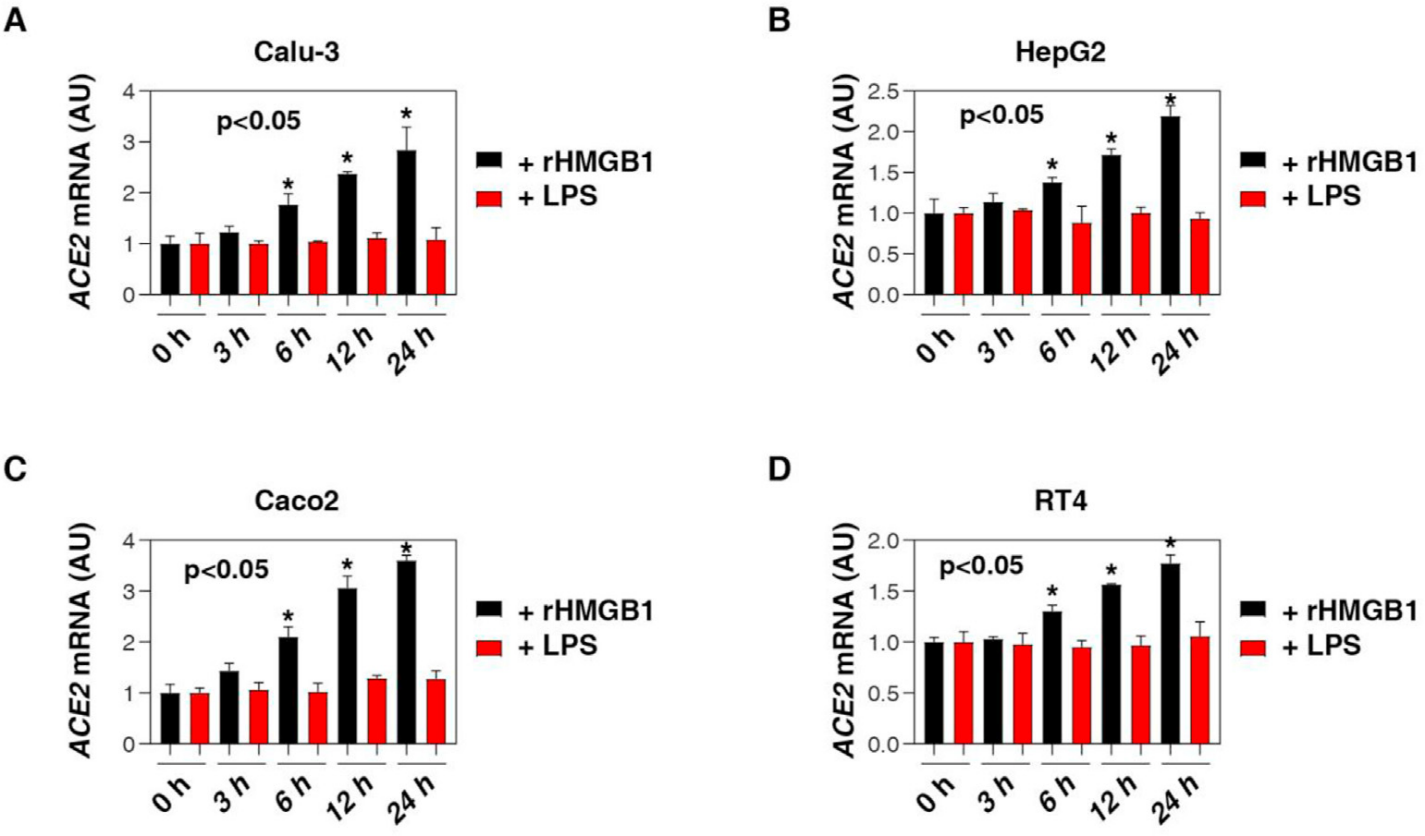
**Recombinant human HMGB1 induces ACE2 expression.** Q-PCR analysis of ACE2 mRNA in indicated Calu-3 (A), HepG2 (B), Caco2 (C) and RT4 (D) cells following treatment with human HMGB1 protein (200 ng/ml) or low-dose LPS (1 ng/ml) for 3–24 h (n = 3, *ANOVA* test). Data represent the mean ± SD of three independent experiments, each performed in triplicate, and are presented relative to control.

The serum assays of HMGB1 in COVID-19 patients were only a preliminary step for the subsequent *in vitro* studies of HMGB1 function. Next, we studied the pathogenic role of extracellular HMGB1 in the regulation of the expression of ACE2, the primary receptor for SARS-CoV-2 and related viruses [2]. Higher ACE2 expression, especially in lung epithelial cells, may lead to increased susceptibility and severity of SARS-CoV-2 infections [2]. Conversely, blocking the expression and activity of ACE2 can limit SARS-CoV-2-induced infection and lung injury [2]. In addition to the well-known inflammatory activity of HMGB1, we found that the exogenous human HMGB1 protein (“rHMGB1”, purity: >95%; endotoxin level: <0.10 EU per 1 μg) time-dependently induced the upregulation of *ACE2* mRNA in Calu-3 cells, a human lung epithelial cell line (Figure 2A). In contrast, low-dose lipopolysaccharides (LPS) failed to induce *ACE2* mRNA expression in Calu-3 cells (Figure 2A), suggesting that the activity of rHMGB1-induced ACE2 expression does not depend on endotoxin contamination. In addition to the lungs, ACE2 can also be expressed in other tissues, such as the small intestine, testes, liver, and kidney [1]. Similarly, rHMGB1 (but not low-dose LPS) also induced the upregulation of *ACE2* mRNA in HepG2 (a human liver cell line), Caco2 (a human intestine cell line), and RT4 (a human urinary bladder cell line) cells (Figure 2C, D). These findings indicate that HMGB1 has a broad role in inducing ACE2 expression in various tissue cells.

### 3.3 AGER is required for HMGB1-induced ACE2 expression in lung epithelial cells

**Figure 3:**
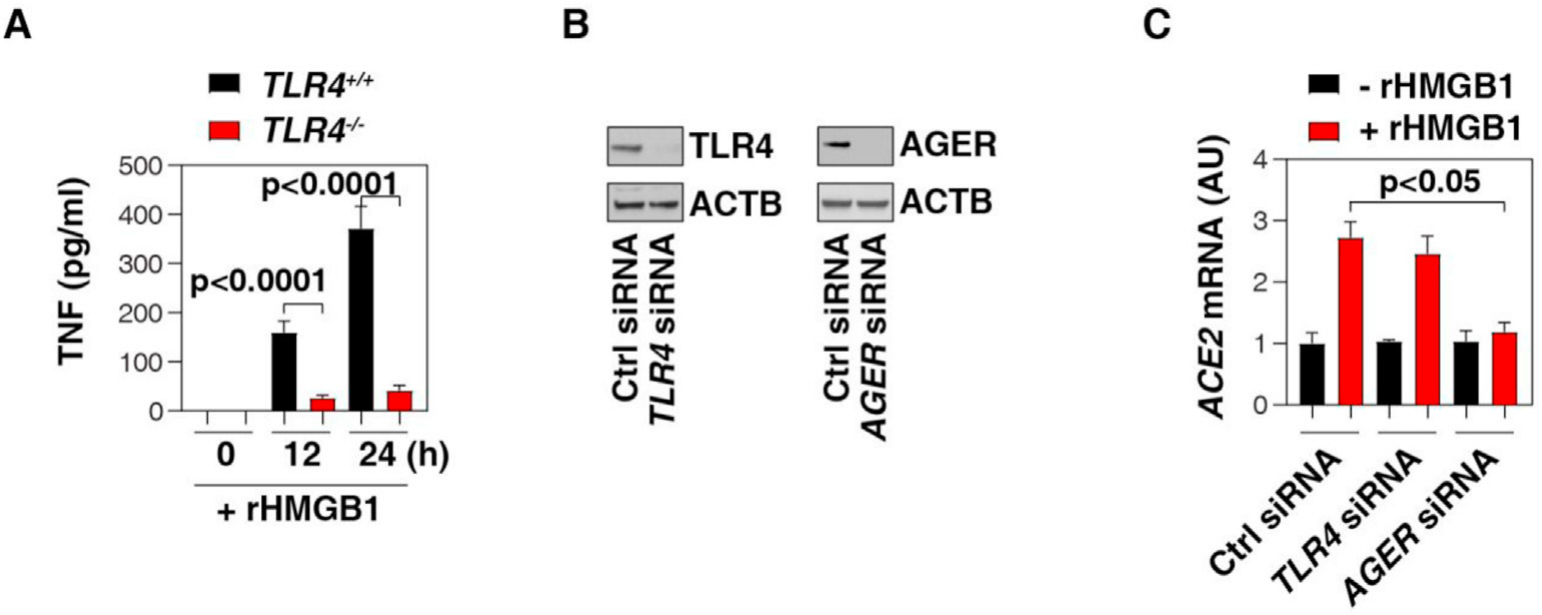
**AGER is required for HMGB1-induced ACE2 expression in lung epithelial cells.** (A) ELISA analysis of TNF release in indicated human monocytes (THP1) following treatment with human HMGB1 protein (200 ng/ml) for 24 h (n = 3, *t* test). (B) Western blot analysis of protein expression in human lung epithelial cells (Calu-3) after siRNA-mediated depletion of TLR4 or AGER. (C) Q-PCR analysis of ACE2 mRNA in indicated Calu-3 cells following treatment with human HMGB1 protein (200 ng/ml) for 24 h (n = 3, *t* test). Data represent the mean ± SD of three independent experiments, each performed in triplicate, and are presented relative to control.

When HMGB1 is released from necrotic cells following infections or tissue damage, it will elicit a TLR4-dependent cytokine storm (largely TNF, IL-6, IL-1, and IL-10) in immune cells (e.g., monocytes, macrophages, neutrophils, and lymphocytes) [10]. We consistently found that rHMGB1-induced TNF release was blocked in TLR4^−/−^ THP1 cell (a human monocytic cell line) (Figure 3A). Unlike TLR4, the lung expression of AGER is mainly localized to alveolar epithelial cells and plays an important role in various lung diseases that include viral infections [24]. Of note, siRNA-mediated depletion of *AGER*, rather than *TLR4*, diminished HMGB1-induced *ACE2* mRNA expression (Figure 3B, C). These findings indicate that AGER is required for HMGB1-induced ACE2 expression in lung epithelial cells. In contrast, TLR4 may contribute to the cytokine release (e.g., TNF) induced by HMGB1 in immune cells, which is also related to COVID-19.

### 3.4 Pharmacological inhibition of HMGB1-AGER signaling limits ACE2 expression

**Figure 4:**
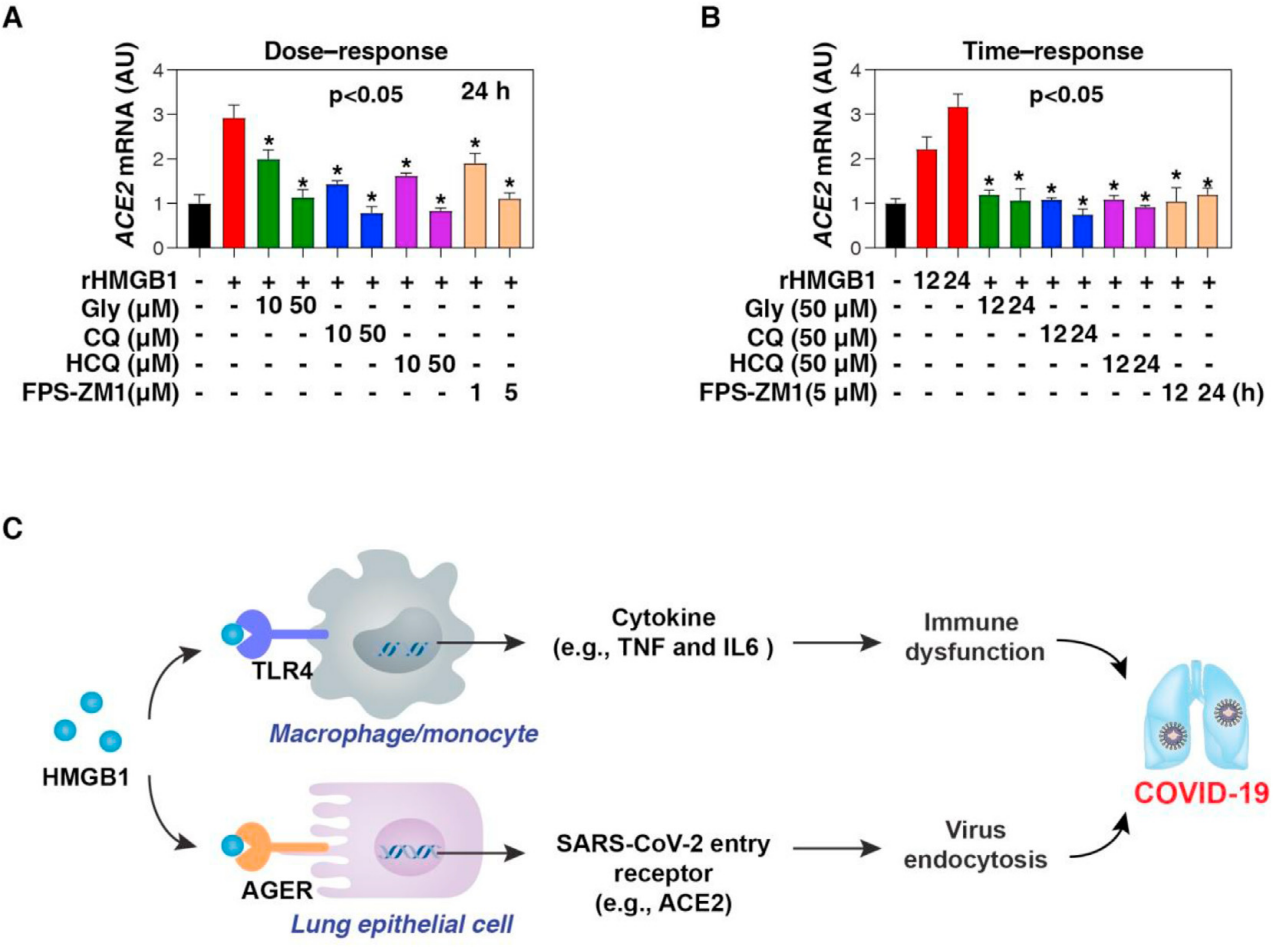
**Pharmacological inhibition of HMGB1-AGER signaling limits ACE2 expression.** (A, B) Q-PCR analysis of ACE2 mRNA in indicated Calu-3 cells following treatment with human HMGB1 protein (200 ng/ml) in the absence or presence of glycyrrhizin (Gly), chloroquine (CQ), hydroxychloroquine (HCQ), and AGER inhibitor FPS-ZM1 for 12 or 24 h (n = 3, ∗P < 0.05 versus HMGB1 group, *ANOVA* test). (C) Schematic depiction of the role of HMGB1 release in COVID-19. HMGB1 may be involved in COVID-19 through at least two mechanisms: one is TLR4-mediated cytokine storm in immune cells (e.g., macrophages and monocytes), and the other is AGER-mediated ACE2 expression in alveolar epithelial cells. Data represent the mean ± SD of three independent experiments, each performed in triplicate, and are presented relative to control.

We also evaluated the efficacy of several HMGB1 inhibitors in the suppression of ACE2 expression. We focused on glycyrrhizin and chloroquine as they show strong activity in blocking extracellular HMGB1 function under various inflammatory conditions, including experimental models of sepsis and septic shock [25, 26]. As a direct HMGB1 inhibitor [27], glycyrrhizin is the main active ingredient of licorice root and has been used in traditional medicine to treat inflammatory and viral diseases [28], including SARS-CoV [29]. In some small clinical trials, the antimalarial drug chloroquine or the derivative hydroxychloroquine is effective in patients with COVID-19 [30]. However, some studies have shown that hydroxychloroquine is ineffective in some patients, partly due to its cardiotoxicity [31], although more clinical trials are ongoing. We found that glycyrrhizin, chloroquine, hydroxychloroquine, and AGER inhibitor FPS-ZM1 [32] all dose- or time-dependently blocked HMGB1-induced *ACE2* mRNA expression in Calu-3 cells *in vitro* (Figure 4A, B).

## 4 Discussion

COVID-19 is a life-threatening viral infection caused by the host's abnormal response to SARS-CoV-2, which is closely related to sepsis and septic shock [1]. Thus, elucidating the pathogenesis of SARS-CoV-2-mediated sepsis is essential for the development of new treatments and drugs. In this study, we demonstrated the direct role of sepsis mediator HMGB1 in promoting ACE2 expression in alveolar epithelial cells, which may form a positive feedback to accelerate viral infection and host damage [18]. These data support the idea that DAMPs are important targets for infectious diseases including COVID-19.

DAMPs are endogenous molecules, including proteins and non-proteins, that can be released or secreted by various stresses (e.g., pathogen infection, oxidative damage, and metabolic crisis) [9]. Among them, HMGB1 is a key DAMP, which plays a pathological role in various infections and sterile inflammation by activating innate and adaptive immunity [11, 12]. In fact, elevated serum HMGB1 levels are associated with a variety of diseases and can be used as biomarkers to predict disease progression, including viral diseases [10]. Consistent with this idea, our current study shows that compared with healthy controls, the serum HMGB1 levels in some severe COVID-19 patients are significantly increased, indicating that there may be a pathological link between HMGB1 and COVID-19. More detailed analysis in large patient cohorts with additional clinical and laboratory observations would be necessary to support this causal relationship.

Moreover, we demonstrated the direct role of HMGB1 in promoting ACE2 expression in cultured human lung epithelial cells via AGER (but not TLR4) expression. Pattern recognition receptors can recognize a variety of molecular patterns, thereby inducing receptor-dependent responses. After release, extracellular HMGB1 can mainly bind to TLRs or AGER in immune cells and non-immune cells, thereby activating and inducing the expression of different genes [10]. Our current study indicates that AGER instead of TLR4 is responsible for extracellular HMGB1-mediated ACE2 expression in alveolar epithelial cells, although the downstream transcription factor regulating ACE2 expression is unclear. These findings also support the previous consensus on the key pathological role of AGER in lung diseases [33].

Importantly, our study highlights that HMGB1 inhibitors or AGER inhibitors can prevent SARS-CoV-2 infection by limiting the upregulation of ACE2. In addition to vaccines, direct treatment strategies may include the development of reagents to block the interaction between SARS-CoV-2 and the host ACE2 receptor, the generation of oligonucleotides targeting the SARS-CoV-2 RNA genome, and administration of convalescent plasma (liquid portion of blood collected from patients recovered from COVID-19) [1]. Reusing existing drugs may provide a faster alternative to COVID-19. Our study indicates that clinically available drugs (e.g., glycyrrhizin, chloroquine, and hydroxychloroquine) may limit the release and activity of HMGB1 during SARS-CoV-2 infection, although the clinical trials of chloroquine and hydroxychloroquine for COVID-19 are still uncertain due to their side effects on the heart [30, 34]. Other plant-derived natural products (e.g., nicotine, epigallocatechin gallate, tanshinone, chlorogenic acid, resveratrol, emodin-6-O-β-D-glucoside, rosmarinic acid, isorhamnetin-3-O-galactoside, persicarin, forsythoside B, acteroside, and shikonin) can also inhibit HMGB1-related inflammation and immune dysfunction in various severe infections [35], and are likewise promising drug candidates for the treatment of patients suffering from COVID-19.

In summary, HMGB1 may be involved in COVID-19 through at least two mechanisms: one is TLR4-mediated cytokine storm in immune cells, and the other is AGER-mediated ACE2 expression in alveolar epithelial cells (Figure 4C). It is still interesting to study how the integrated network between DAMPs and cytokines promotes COVID-19. Although the current preliminary *in vitro* studies indicate that HMGB1 has a potential role in COVID-19, novel animal models of SARS-CoV-2 infection will undoubtedly be of great value for further evaluation of the use of HMGB1 inhibitors in the treatment of COVID-19.

## Declarations

### Author contribution statement

R. Chen: Conceived and designed the experiments; Performed the experiments; Analyzed and interpreted the data; Contributed reagents, materials, analysis tools or data; Wrote the paper.

Y. Huang, J. Quan and J. Liu: Performed the experiments; Analyzed and interpreted the data; Contributed reagents, materials, analysis tools or data.

H. Wang, T. Billiar, M. Lotze, H. Zeh and R. Kang: Analyzed and interpreted the data; Wrote the paper.

D. Tang: Conceived and designed the experiments; Analyzed and interpreted the data; Wrote the paper.

### Funding statement

This research did not receive any specific grant from funding agencies in the public, commercial, or not-for-profit sectors.

### Data availability statement

Data included in article/supplementary material/referenced in article.

### Declaration of interests statement

The authors declare no conflict of interest.

### Additional information

No additional information is available for this paper.
